# Household and area-level social determinants of multimorbidity: a systematic review

**DOI:** 10.1136/jech-2020-214691

**Published:** 2020-11-06

**Authors:** Elizabeth Ingram, Sarah Ledden, Sarah Beardon, Manuel Gomes, Sue Hogarth, Helen McDonald, David P Osborn, Jessica Sheringham

**Affiliations:** 1 Department of Applied Health Research, University College London, London, UK; 2 Division of Psychiatry, University College London, London, UK; 3 London Boroughs of Camden and Islington, London, UK; 4 London School of Hygiene and Tropical Medicine, London, UK; 5 Camden and Islington NHS Foundation Trust, London, UK

**Keywords:** Deprivation, Health inequalities, Public health, Social inequalities, Epidemiology of chronic non communicablenon-communicable diseases

## Abstract

**Background:**

No clear synthesis of evidence examining household and area-level social determinants of multimorbidity exists. This study aimed to systematically review the existing literature on associations between household and area-level social determinants of health (SDoH) and multimorbidity prevalence or incidence in the general population.

**Methods:**

Six databases (MedLine, EMBASE, PsychINFO, Web of Science, CINAHL Plus and Scopus) were searched. The search was limited to peer-reviewed studies conducted in high-income countries and published in English between 2010 and 2019. A second reviewer screened all titles with abstracts and a subset of full texts. Study quality was assessed and protocol pre-registered (CRD42019135281).

**Results:**

41 studies spanning North America, Europe and Australasia were included. Household income and area-level deprivation were the most explored with fairly consistent findings. The odds of multimorbidity were up to 4.4 times higher for participants with the lowest level of income compared with the highest level. Those living in the most deprived areas had the highest prevalence or incidence of multimorbidity (pooled OR 1.42, 95% CI 1.41 to 1.42). Associations between deprivation and multimorbidity differed by age and multimorbidity type. Findings from the few studies investigating household tenure, household composition and area-level rurality were mixed and contradictory; homeownership and rurality were associated with increased and decreased multimorbidity, while living alone was found to be associated with a higher risk of multimorbidity and not associated.

**Conclusion:**

Improving our understanding of broader social determinants of multimorbidity—particularly at the household level—could help inform strategies to tackle multimorbidity.

## INTRODUCTION

Multimorbidity is one of the greatest challenges for health and care systems worldwide.^1^ Broadly defined as the co-occurrence of multiple chronic conditions within the same individual,^2^ multimorbidity is now the norm internationally, not the exception.^3^ Indeed, approximately one-third of UK primary care patients has two or more long-term conditions.^4^ This is projected to increase dramatically over the coming decade.^5^

Multimorbidity challenges health and care systems because care must, by definition, cross organisational and sectoral boundaries.^6 7^ This requires a radical shift in approach from a fragmented medical model of illness, centred on specific disorders, to a more holistic view of health. There are also calls for approaches focused on preventing multimorbidity or curtailing multimorbidity progression that could minimise the future burden on the system.^1^ Such shifts in approach require an understanding of the broader factors associated with multimorbidity.

While multimorbidity is often framed as a health issue, it is greatly influenced in extent and nature by social determinants of health (SDoH). There are multiple conceptualisations of SDoH; nevertheless, a hierarchical division of individual, household and area-level social factors is common to many.^8–10^ Individual social determinants (SD) of multimorbidity are well established—prevalence is greater among ethnic minorities and individuals with fewer educational qualifications.^11–14^ However, understanding of household and area-level SD is limited with most primary research focusing on area-level deprivation indices. At present, no clear synthesis of evidence examining household and area-level SD of multimorbidity exists.^11 15–18^

This review aimed to systematically identify, critically appraise and synthesise the existing literature on associations between household and area-level SDoH and multimorbidity prevalence or incidence in general populations of high-income countries (HICs). We also aimed to investigate how associations differ with age, gender and ethnicity. Better understanding of SD of multimorbidity could inform equitable prevention and intervention strategies.

## METHODS

This review was conducted following PRISMA guidelines^19^ and the protocol prospectively registered with PROSPERO (CRD42019135281).

### Eligibility criteria for inclusion

Studies were included if in English, conducted in HICs and published between 1 January 2010 and 10 January 2019. The former date restriction coincides with the publication of The Marmot Report, which raised the profile of SDoH in England.^20^ We excluded studies conducted solely with institutionalised individuals as SD of multimorbidity may differ between institutional and community settings.^21^ We excluded studies conducted with solely young people (<18 years) as prevalence is low for this group^22 23^ (table 1).

**Table 1:** Inclusion and exclusion criteria

	Inclusion	Exclusion
Population	Participants from the general population and assessed for the presence of multiple chronic conditions (multimorbidity).	Participants initially selected based on the presence of index diseases (ie, studies of comorbidity). Participants from solely institutionalised care settings (eg, nursing homes). Participants solely young people (age <18 years).
Exposure	Study exposure(s) included at least one household or area-level SDoH that aligns with factors from the World Health Organisation (WHO) Commission on SDoH (CSDH) Framework^9^ and the idea that SDoH are ‘causes of the causes’ of ill-health^20^ (eg, household income or area-level deprivation).	Study exposure(s) include individual SDoH only (eg, ethnicity). Study exposure(s) are direct “causes” of ill-health, such as health behaviours (eg, smoking), or are factors associated with the health system itself (eg, access to services).
Comparator	Study reports comparator group(s) for SDoH exposure(s) (eg, what is the prevalence of multimorbidity for those in the lowest vs the highest household income groups).	Study does not report a comparator group for SDoH exposure(s).
Outcome	Assess multimorbidity burden (prevalence or incidence studies).	Assignment to multimorbidity patterns or trajectories. Measures of multimorbidity severity (eg, indices used weighted by disease severity).
Study design	Peer-reviewed studies of quantitative research designs (cross-sectional and longitudinal).	Systematic reviews, meta-analyses and qualitative research (citations of relevant reviews searched).

### Search strategy

The following six databases were searched: MedLine, EMBASE, PsychINFO, Web of Science, CINAHL Plus and Scopus. Terms relating to multimorbidity, specific SDoH and household or area were combined using Boolean language. We drew on published frameworks and previous literature to develop SDoH search terms.^8–10 24–27^ Terms were initially developed in MedLine (online appendix 1) and adapted for each database. After the initial search, we added the MeSH term ‘comorbidity’ into our MedLine search to examine if any studies had been missed through excluding the term ‘comorbidity’ and its linguistic variations. A combination of forward and backward citation searching, and searching citations of relevant reviews, was used to identify further studies.

10.1136/jech-2020-214691.supp1 Supplementary data

EI and SL independently screened titles and abstracts of all records from database searches. A third reviewer independently screened 100 randomly selected records. EI screened all full texts and SL a subset (20%). All relevant data were extracted by EI using a pre-piloted form including study characteristics, definitions of exposures and outcomes, and findings (online appendix 2). The authors were contacted if data extraction was incomplete. Inter-rater reliability was calculated using Kappa statistics and differences resolved by discussion.

10.1136/jech-2020-214691.supp2 Supplementary data

### Quality assessment

Study quality was assessed within four domains: selection bias, information bias for exposure and outcome, and confounding (online appendix 3). Non-interventional studies are rarely at low overall risk of bias and reporting by domain allows comparison of the main sources of bias across studies.^28^ Each study was assigned high, medium, low or unclear ratings for each domain, to separate study quality from reporting quality.^29 30^ Criteria were specific to the study and informed by existing checklists.^28 31^ Risk of selection bias was assessed by comparing sample demographics to census data when possible. Studies where risk of bias was high across two or more domains were deemed low quality. Studies where risk of bias was mixed or medium across all domains were deemed moderate. Studies with a low risk of bias across two or more categories, with no high risk of bias across any domains, were deemed high quality. EI completed all assessments and SL a random subset (20%). Quality assessments were used to provide insight into the overall quality of evidence in this field, rather than to exclude or rank studies. Assessments were also used to explore any associations between study results and quality assessments.

10.1136/jech-2020-214691.supp3 Supplementary data

### Data synthesis

Findings were narratively synthesised given the diverse exposures, outcomes and study methodologies. Studies were too heterogeneous to allow a meta-analysis of findings. Available data were pooled for studies investigating area-level deprivation to calculate overall multimorbidity prevalence in deprivation quintiles.

## RESULTS

### Study selection and characteristics

**Figure 1:**
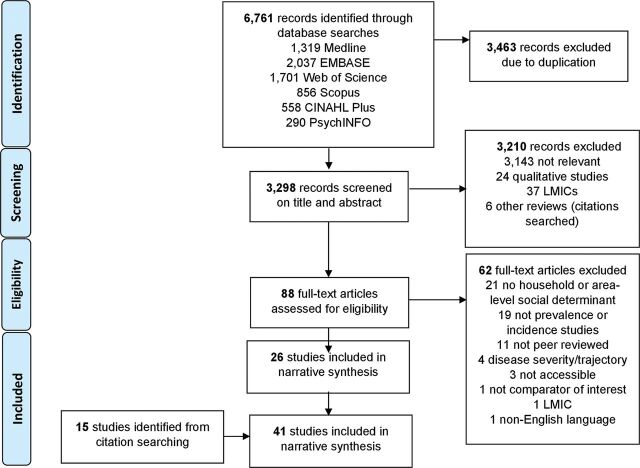
PRISMA flow diagram.

Forty-one studies were included (figure 1). Inter-rater reliability was good for title and abstract screening (κ=0.71), and full-text (κ=0.77).^32^

Online appendix 4 details key study characteristics. Studies were conducted in a range of countries, the most common being Canada, England and Spain. Sample sizes ranged from 232 to 13 581 191. Twenty-five studies included participants from across the life-course, while nine focused on adults aged 50 and over. Household SDoH included measures of household income, tenure and composition, self-reported by participants in all studies. Area-level SDoH included measures of socioeconomic deprivation and rurality, the former measured using validated indices (16/17 studies) and polling data (one study).

10.1136/jech-2020-214691.supp4 Supplementary data

### Defining and measuring multimorbidity

Most studies (34/41) defined multimorbidity as two or more conditions taken from a pre-specified list of ‘long-term’ or ‘chronic’ conditions. Eight also used three or more conditions as the cut-off and seven studies used a count of conditions as one outcome. Six studies examined if associations differed with multimorbidity type: multimorbidity specifically comprising physical and mental health conditions (physical–mental multimorbidity), physical conditions (physical-only multimorbidity) and mental health conditions (mental-only multimorbidity).

Across the 41 studies, the number of conditions included on the pre-specified list ranged from five conditions to 146 diagnostic clusters defined using O’Halloran’s criteria for chronicity.^33^ Thirty-six of 41 studies included a mix of chronic physical and mental health conditions,^4 22 34–67^ while four included physical conditions only.^68–71^ One study was unclear about the conditions included.^72^ To determine the presence of conditions, 17 studies used self-reported participant data, 17 screened electronic health records (EHRs), and six used a combination of the two. One study did not detail how they identified multimorbidity presence^70^ (online appendix 4).

### Study results

**Household income (n=15):** Thirteen studies consistently found multimorbidity prevalence or incidence was markedly and negatively associated with household income and, of all SDoH investigated, associations were consistently strongest for household income.^39–41 43 48 53 60–62 65 66 68 69^ Higher quality studies reported comparatively small estimated effect sizes, for example, Agborsangaya *et al* reported that an annual household income <$30 000 CAD was associated with a 2.39-fold increase in multimorbidity prevalence (95% CI 1.72 to 3.33) compared with ≥$100 000 CAD, after multiple adjustments.^62^ In contrast, Roberts *et al*—a lower quality study—reported chances of multimorbidity 4.4 times higher for participants with the lowest level of income compared with the highest in multivariate analyses (OR 4.4, 95% CI 3.6 to 5.5).^53^

Two further studies—of low and moderate quality, respectively—examined problems managing household income and reported mixed results.^52 59^ Verest *et al* reported those with ‘lots of problems’ were over 5 times likely to have multimorbidity compared with those with ‘no problems’ (OR 5.36, 95% CI 4.88 to 5.88). Inequalities were similar by gender and ethnicity.^59^ In contrast, Prazeres *et al* found no evidence of an association when screening EHRs.^52^

See table 2 for key results and quality assessments for these 13 studies, and online appendix 4 for more details on study characteristics.

**Table 2:** Key results and quality assessments for studies investigating household income (n=13)

First author (Year)	Key results				Risk of Bias¶			
*Household income*	Association between SDoH and MM?	Value (95% CI, p value)	Comparator	Adjusted for…	Selection	Information (Exposure)	Information (Outcome)	Confounding
Agborsangaya (2012)^61^	Yes	OR 2.39**(1.72–3.33)	Annual household income <$30 k vs ≥$100 k CAD	Age, sex, education, living with children	H	M	M	L
Agborsangaya (2013)^62^	Yes	OR 2.9 (2.2–3.7)	Annual household income <$30 k vs ≥$100 k CAD	Age, sex, education, obesity	H	H	M	L
Chung (2015)^65^	Yes	OR 1.52 (1.39–1.66, p<0.001)	Monthly income <4 k vs >40 k HKD	Age, gender, education, housing, employment	H	M	M	L
Hayek (2017)^68^	Yes	PRR 1.7 (1.2–2.5, p=0.005)	Monthly income ≤$2 k vs >$4 k USD	Unclear	U	H	H	U
Johnson-Lawrence (2017)^69^	Yes	OR 1.45 (1.38–1.53)	Lowest income tertile vs highest	Age, gender, ethnicity, education, interview year, region, marital status, last doctor visit, employment, home ownership	U	M	H	L
Katikireddi(2017)^39^	Yes	OR 1.53 (1.25–1.87, p<0.05)	Lowest income† tertile vs highest	Age, age^2^, age^3^, sex, cohort, prior multimorbidity, time between waves and sex*cohort interaction	M	M	M	L
Ki(2017)^40^	Yes	OR 3.48* (3.20–3.78)	“Poor” (less than half the median annual household income†) vs “non-poor”	No adjustment	U	H	M	H
Laires(2018)^41^	Yes	OR 2.16* (1.95–2.40)	Lowest income† quintile vs highest	No adjustment	L	H	M	H
Lebenbaum(2018)^66^	Yes	OR 0.57 (0.52–0.62, p<0.001)	Highest income† quintile vs lowest	Age, age,^2^ sex, marital status, immigration status, education, rurality, homeownership, smoking, alcohol use	L	M	H	L
Lujic (2017)^43^	Yes	OR 0.58‡ (95% CI 0.52 to 0.66)	Income >$70 k vs <$20 k CAD	Age, sex	H	M	M	M
Neilsen (2017)^48^	Yes	OR 1.44 (1.32–1.59, p<0.05)	Lowest income tertile vs highest	Age, sex, education	U	H	M	L
Prazeres (2015)^52^	No	OR 0.8§ (0.5–1.1, p=0.182)	‘Some monthly income left over’ vs ‘Not enough monthly income to make ends meet’	Age, sex, marital status, education, professional status, residence area, living arrangement	H	M	L	L
Roberts (2015)^53^	Yes	OR 4.4 (3.6–5.5)	Lowest income quintiles vs highest	Age, sex, household education, Aboriginal status, activity level smoking, stress, blood pressure, obesity	H	M	H	M
Schäfer(2012)^60^	Yes	−0.27 conditions(−0.47 to −0.08, p=0.005)	Change per unit on income† scale (one unit=one of steps: €400 to €1100 to €3000 to €8100 net income per month)	Age, gender, marital status, job autonomy, household composition, income	H	M	L	U
Verest(2019)^59^	Yes	OR 5.36*, †† (4.88–5.88)	“Lots of problems” managing money vs “no problems”	No adjustment	H	H	M	H

*OR calculated from data reported in paper.

†Income equivalised to account for number and/or age of residents in household.

‡Based on self-reported health data. Findings consistent across hospital and medication health data.

§Multimorbidity defined as ≥2 chronic conditions.

¶H, High; M, Medium; L, Low; U, Unclear.

**Inequalities greater for ages 25–44.

††Inequalities greater for women and similar by ethnicity group.

**Household composition (n=7):** Four studies measured household composition as living alone vs cohabiting and three studies measured it as living alone, living with various family members or living in other situations (including care homes).

Four cohort studies of older adults (50–84) reported mixed findings on the risk of living alone vs cohabiting.^37 45 47 70^ Two high-quality studies found living alone increased chances of multimorbidity vs living with others,^37 70^ for example, Cantarero-Prieto *et al* found living alone increased chances of multimorbidity by 20% (OR 1.20, 95% CI 1.04 to 1.39, p<0.05).^70^ Whereas two other studies—high and moderate quality—found no evidence, living alone was associated with multimorbidity incidence.^45 47^ Differences in study characteristics such as methods of ascertaining multimorbidity presence could not explain these mixed findings.

Of the three studies with alternative measures of composition, one moderate-quality cross-sectional study found that odds of multimorbidity were over 2 times greater if not living with children vs living with children (OR 2.11, 95% CI 1.60 to 2.78; adjusted for age).^61^ Two further moderate-quality studies (one included solely older adults) found no evidence of any associations with multimorbidity when living alone was compared with living as a couple, with family/others or living in situations such as care homes.^52 60^

See table 3 for key results and quality assessments for these seven studies, and online appendix 4 for more details on study characteristics.

**Table 3:** Key results and quality assessments for studies investigating household composition (n=7), household tenure (n=4) and household rurality (n=7), structured per social determinant

					Risk of Bias§			
First author (Year)				Key results	Selection	Information (Exposure)	Information (Outcome)	Confounding
***Household composition***								
Agborsangaya (2012)^61^	Yes No	OR 2.11¶ (1.60–2.78) Data not available	Living with children vs not living with children Living with adults vs not living with adults	Age, sex, education and household income	H	M	M	L
Cantarero-Prieto (2018)^70^	Yes	OR 1.20 (1.04–1.39, p<0.05)	Living alone vs cohabits	Unclear	U	U	M	U
Henchoz (2019)^37^	Yes	OR 1.40* (1.21–1.61)	Living alone vs cohabits	No adjustment	U	M	M	M
Melis (2014)^45^	No	OR 1.34 (0.60–3.01)	Living alone vs cohabits	No adjustment	U	M	L	H
Mounce (2018)^47^	No	HR 0.93 (0.71–1.21, p=0.580)	Living alone vs cohabits	Baseline age, sex, total wealth, educational attainment, health behaviours, social detachment and locus of control	U	M	M	L
Prazeres (2015)^52^	No	OR 1.4§ (0.9–2.3, p=0.182) OR 1.0§ (0.6–1.7, p=0.985) OR 1.3§ (0.7–2.6, p=0.410)	Living as a couple vs alone Living as extended family vs alone Living in other situation (inc. care home) vs alone	Age, sex, marital status, education, professional status, residence area, living arrangement	H	M	L	L
Schäfer (2012)^60^	No	−0.10 conditions (−0.42–0.23, p=0.562) 0.24 conditions (−0.14–0.62, p=0.210) −0.01 conditions (−0.59–0.57, p=0.231)	Living at home with spouse vs home alone Living at home with family members or others vs home alone Living in assisted living or retirement home vs home alone	Age, gender, marital status, job autonomy, household composition, income	H	M	L	U
***Household tenure***								
Chung(2015)^65^	Yes	OR 1.17 (1.11–1.24, p=0.003) OR 1.19 (1.09–1.29, p=0.041) OR 1.11 (1.05–1.18, p=0.070)	Homeowner vs public (social) housing Private renting vs public (social) housing Subsidised housing vs public (social) housing	Age, gender, education, housing, employment	H	M	M	L
Johnson-Lawrence (2017)^69^	Yes	OR 1.19 (1.15–1.24)	Renters vs homeowners	Age, gender, ethnicity, education, interview year, region, marital status, last doctor visit, employment, household income	U	M	H	L
Lebenbaum(2018)^66^	Yes	OR 0.82 (0.78–0.87, p<0.001)	Homeowners vs non-homeowners	Age, age^2,^ sex, marital status, immigration status, education, rurality, homeownership, smoking, alcohol use	L	M	H	L
Schäfer (2012)^60^	No	−0.13 conditions (−0.30–0.05, p=0.148)	Homeowners vs non-homeowners	Age, gender, marital status, job autonomy, household composition, income	H	M	L	U
***Rurality***								
Cantarero-Prieto (2018)^70^	No	OR 0.92 (0.93–1.03, p>0.1)	Living in rural vs non-rural areas	Unclear	U	U	M	U
Foguet-Boreu (2014)^36^	Yes	OR 1.04*,** (1.03–1.05)	Living in rural (<10 000 inhabitants and/or population density <150 people/km^2^) vs non-rural areas	Unadjusted	U	L	L	U
Lebenbaum (2018)^66^	No	OR 0.98 (0.93–1.02, p=0.323)	Rural vs non-rural areas	Age, age^2,^ sex, marital status, immigration status, education, rurality, homeownership, smoking, alcohol use	L	M	H	L
Lujic (2017)^43^	Yes	OR 1.14† (1.03–1.26)	Living in remote/very remote areas (vs major cities)	Age and sex	H	M	M	M
Prazeres (2015)^52^	No	OR 1.0‡ (0.8–1.3, p=0.746)	Living in rural vs urban areas	Age, sex, marital status, education, professional status, residence area, living arrangement	H	M	L	L
Roberts (2015)^53^	Yes	OR 1.1 (1.0–1.3)	Living in rural vs urban areas	Age, sex, household education, household income, Aboriginal status, activity level, smoking, stress, blood pressure, obesity	H	M	H	M
Ryan (2018)^54^	Yes	OR 0.85* (0.85–0.86)	Living in rural (<10 000 inhabitants) vs non-rural areas	Age-sex standardised	L	L	L	M

*OR calculated from data reported in paper.

†Based on self-reported health data. Findings consistent across hospital and medication health data.

‡Multimorbidity defined as ≥2 chronic conditions.

§H, High; M, Medium; L, Low; U, Unclear.

¶Associations greater for 65+.

**Inequalities similar with gender and greater≥45 years.

**Household tenure (n=4):** Findings from studies investigating tenure were mixed and hard to compare given different reference groups and comparators; two studies compared homeowners and non-homeowners, one compared renters with homeowners and one compared social housing residents with homeowners, private renters and subsidised housing residents.^60 65 66 69^ All four were of moderate quality. Lebenbaum *et al* found the odds of multimorbidity decreased by 18% for homeowners compared with non-homeowners (OR 0.82, 95% CI 0.78 to 0.87, p<0.001),^66^ whereas Johnson-Lawrence *et al* reported 19% higher odds for renters vs homeowners (OR 1.19, 95% CI 1.15 to 1.24).^69^ In contrast, Schäfer *et al*—the only cohort study—found no evidence of an association between homeownership and count of conditions in older adults.^60^ One study found that, compared with social housing residents, homeowners and private renters had 17% (OR 1.17, 95% CI 1.11 to 1.24, p=0.003) and 19% (OR 1.19, 95% CI 1.09 to 1.29, p=0.041) higher odds of multimorbidity, respectively.^65^ Differences in study characteristics could not explain mixed findings.

See table 3 for key results and quality assessments for these four studies, and online appendix 4 for more details on study characteristics.

**Household determinants in childhood (n=5):** Two studies examined associations between paternal social class at birth and multimorbidity. Findings were mixed. One higher quality study from Johnston *et al* found lower paternal social class at birth was associated with increased multimorbidity in middle age.^72^ Conversely, one study of lower quality reported no association.^38^

Two studies investigated associations between self-reported childhood financial hardships and multimorbidity and, again, findings were mixed.^37 71^ One higher quality study found no evidence of an association,^37^ whereas one fairly low-quality study found evidence that the number of chronic conditions for those reporting hardships was 1.19 times that of those not reporting hardships (95% CI 1.07 to 1.32, p<0.001).^71^

One further study, moderate in quality, found the odds of multimorbidity increased by 40% among those who had experienced household dysfunction during childhood (eg, parental divorce) in multivariate analyses (OR 1.4, 95% CI 1.1 to 1.7, p<0.05).^56^

**Household primary language and education (n=2):** One moderate-quality Australian study found associations between living in a household where English was the first language and multimorbidity prevalence differed depending on the source of health information.^43^ One lower quality study found higher odds of multimorbidity for participants living in households where no residents had completed high school compared with post-secondary school education (OR 1.8, 95% CI 1.6 to 2.1, adjusting for age and sex).^53^

**Household rurality (n=7):** Studies that investigated associations between residing in rural vs non-rural/urban areas and multimorbidity reported mixed results.^36 43 52–54 66 70^ Only two studies were high quality and provided clear rurality definitions; these both suggested odds of multimorbidity decreased with increased rurality.^36 54^ Conversely, two studies, one low^53^ and one moderate quality^43^ reported greater odds of multimorbidity with increased rurality. Three further studies found no evidence of any association.^52 66 70^ Aside from study quality, differences in study characteristics could not explain these mixed findings.

See table 3 for key results and quality assessments for these seven studies, and online appendix 4 for more details on study characteristics.

**Figure 2:**
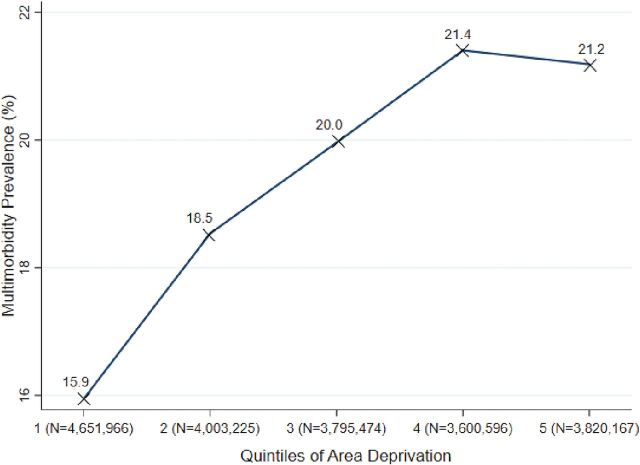
Multimorbidity prevalence with quintiles of area-level deprivation (1=least deprived and 5=most deprived). Calculations conducted by authors and based on available and relevant pooled data from N=7 out of 17 studies.

**Area-level socioeconomic deprivation (n=17):** Studies that investigated how the socioeconomic situation of participants’ residential area was associated with multimorbidity prevalence or incidence showed fairly consistent findings.^4 22 34 35 39 42 44 46 49–51 54 55 57 63 64 67^ In general, multimorbidity was higher for participants residing in areas of greater deprivation than those living in more affluent areas. Odds of multimorbidity prevalence were 42% higher for participants residing in the most vs the least deprived areas when available data were pooled (OR 1.42, 95% CI 1.41 to 1.42; figure 2). Differences in study quality could not explain differences in reported effect sizes across studies.

Four studies found inequalities with area-level deprivation were greater among women than men.^35 49–51^ Only one low-quality study investigated how associations differed by ethnicity, and they found that inequalities were greater for Pacific vs Maori New Zealand residents.^67^

**Trust in neighbours (n=1):** One fairly low-quality study found that participants who somewhat distrusted their neighbours had increased risk of developing multimorbidity within 11 years compared with those who strongly disagreed with the statement ‘One cannot trust each other here’ (RR 1.13, 95% CI 1.03 to 1.23).^58^

**Older adult populations and older adult sub-group analyses (n=21):** Nine studies included samples of adults solely aged 50 and over.^37 38 45–47 50 60 70 71^ Five of these examined associations between household composition and multimorbidity (results described above).^37 45 47 60 70^ There were no differences in results for studies with older adults only compared with studies that included younger adults.

Thirteen studies examined whether associations differed with subgroups of age.^4 22 35 36 42 44 46 50 51 53 57 61 63^ Estimated effect sizes were greater in younger compared with older adults for household income—for example, Roberts *et al* reported greater odds of multimorbidity amongst 35–49 year olds compared with over 65s for those with the lowest income vs the highest (OR 7.5, 95% CI 4.0 to 13.7 vs OR 2.5, 95% CI 1.8 to 3.5, respectively; see table 2).^53^ However, Agborsangaya *et al* (2012) found that associations between multimorbidity and not living with children (vs living with children) were greater for those ≥65 years of age compared with those 25–44, adjusting for sex and household income (OR 8.45, 95% CI 2.02 to 35.41 vs OR 2.00, 95% CI 1.29 to 3.02, respectively; see table 3).^61^ Ten studies found that differences in multimorbidity prevalence with area deprivation reduced in older age.^4 22 35 42 44 46 50 51 57 63^ Inequalities across deprivation categories were greater in middle age for general multimorbidity,^4 22 35 42 44 51 57 63^ in younger age groups for physical–mental multimorbidity^4 44^ and mental-only multimorbidity,^44^ and in older age for physical-only multimorbidity.^44^ One study examining household rurality reported data showing similar associations in older vs young groups^36^ (see table 3).

Online appendix 2 outlines our full data extraction table for all 41 studies.

## DISCUSSION

This is the first study to systematically review and appraise existing literature on associations between household and area-level SDoH and multimorbidity. Household income and area-level deprivation were the most explored SDs, and findings for these were fairly consistent; odds of multimorbidity were up to 4.4 times higher for those within the lowest level of household income (vs the highest), and prevalence was 1.4 times higher in the most vs the least deprived areas. Other household and area-level SDoH have been underexplored.

### Possible explanations for our findings

Previous research has proposed that household factors are often overlooked in studies exploring SDoH, despite households (or families) influencing physical and mental health through various material and psychosocial factors.^73–75^

In this review, we identified seven studies that investigated household composition and four that investigated household tenure. Composition studies presented mixed results; living alone was associated with increased multimorbidity in two studies and not associated in four. These studies included different reference groups and comparators, making them hard to compare. For example, ill-health greatly drives care home admissions^76^ and therefore comparing ‘living alone’ with either ‘not living alone’ or ‘living in a care home’ would likely be comparing groups in different health, leading to differential associations between household composition and multimorbidity. One further study found living with children (vs not) was associated with increased chances of multimorbidity, and this effect was greater for over 65s. Chronic illness may give rise to older individuals residing with family and may lead younger individuals unable to (or decide not to) have children. Interestingly, none of the included studies examining household composition adjusted for care provision, which can differ considerably for those living with a partner, family or alone^77^ and could plausibly influence the relationship between composition and multimorbidity. Further research should gather data on care provision and adjust accordingly. Unpicking whether social circumstances drive multimorbidity, or vice versa, also requires better designed longitudinal studies. This could aid the targeting of resources for prevention.

Studies investigating household tenure reported contradicting results; homeownership was associated with both increased and decreased chances of multimorbidity. Comparing these results was, again, complicated by different reference groups and comparators; however, study contexts may be more pertinent here. These studies were conducted in Hong Kong, Canada, USA and Germany. The degree of homeownership, and supply and conditions of social housing, may vary across these locations, for example, approximately 45% of the Hong Kong population lived in public housing in 2019 compared with 10% of the German population in 2017.^78 79^ This, plus other social circumstances, could profoundly influence the status and stigma associated with owning, renting, or residing in social housing across geographies and over time, differentially impacting health and associations between tenure and multimorbidity.^80–82^

A minority of studies examined whether associations differed by age, gender or ethnicity. Findings suggest women experience greater inequality in multimorbidity prevalence with area-level deprivation, in line with research highlighting an increase in life expectancy inequality for UK women.^83^ Prevalence with area-level deprivation was also greater for younger populations for physical–mental multimorbidity, unsurprising given the consistently high prevalence of mental ill-health among young, deprived communities.^84 85^ This, however, suggests that studies excluding mental health conditions from multimorbidity definitions or specifying multimorbidity as specifically crossing physical and mental health may report different associations than studies not. Future research should consider physical and mental dimensions of multimorbidity and examine whether associations differ by key demographics. Further avenues for future research should also examine the main explanatory factors, for example, whether individual socioeconomic factors (such as education) or behavioural factors (such as tobacco use) confound or explain any observed associations. This could also aid the development of tailored prevention and intervention strategies.

Lack of consensus around a multimorbidity definition is a consistently raised issue.^86^ In this review, most studies defined multimorbidity as two or more chronic conditions (the most used definition in the literature^86^); however, several also used a cut-off point of three or more or a count of conditions. To ascertain the presence of multimorbidity, the included studies used either self-reported data, data from EHRs or a combination of the two. This hampered effective comparisons of study findings, yet we found no evidence suggesting differences in findings could be explained by differences in multimorbidity definitions or measurement methods. There was also no variation in determinants of multimorbidity by measurement methods used. Consistent definitions of multimorbidity and consistent methods for ascertaining its presence are needed to improve the comparability of findings.

### Strengths and limitations of this review

Strengths of this paper include the systematic inclusion of household SDoH, which has captured studies missed by previous reviews,^11 15–18^ and the careful assessment of each study for risk of four dimensions of bias using pre-specified criteria tailored to the study. Limitations include that we excluded the term ‘comorbidity’ and its linguistic variations from our search despite it being used interchangeably with ‘multimorbidity’.^87^ While this may have missed some relevant literature, a subsequent ad hoc search in MedLine, that included this term, did not identify any additional, relevant hits. A large proportion of our included studies were also identified via citation searching. We believe that this is an intrinsic issue when conducting these types of reviews; in the literature, SDoH are referred to by the determinant of interest (eg, ‘rurality’) and search strategies need to pre-specify terms to search for these, potentially missing relevant studies. We also restricted our search to English-language publications and excluded studies conducted in low- and middle-income countries (LMICs) as the socioeconomic gradient in multimorbidity is reversed in LMICs.^88^

### Implications for policy, research and practice

Household determinants of multimorbidity other than income are often overlooked. Given the comparatively large effect sizes for household compared with area-level SDoH, our study suggests that strategies to tackle multimorbidity should consider household-level factors. There is also a need for additional studies in different geographical contexts to gain a better understanding of the role of household SDoH on multimorbidity. Policies aimed at reducing social inequalities could be important components of strategies to tackle multimorbidity.

**What is already known on this subject**

- Multimorbidity—the co-occurrence of multiple chronic conditions within the same individual—is influenced in extent and nature by social determinants of health (SDoH). Associations between individual SDoH, such as education and individual income, are well documented; prevalence is higher with lower education levels and income. However, no clear synthesis of household and area-level social determinants of multimorbidity exists.

**What does this study add**

- We found that, aside from household income, household social determinants of multimorbidity are underexplored. Given the comparatively large effect sizes for household compared with area-level SDoH, our study suggests that strategies to tackle multimorbidity should consider household-level factors.
