# Supplementary material for: Household and area-level social determinants of multimorbidity: a systematic review
Source: J Epidemiol Community Health. 2020 Nov 6;75(3):232–41. doi: 10.1136/jech-2020-214691 (PMC7892392; doi:10.1136/jech-2020-214691)
Supplement: Supplementary data [file jech-2020-214691supp002.pdf]

Appendix 2: Data extraction table for included studies (n=41)

First Author<br>Year<br>Country	Study Design	Participants	Exposure(s)	Outcome(s)[a]	Key Findings[b]	Risk of Biases[c]			
						Selection	Information (Exposure)	Information (Outcome)	Confounder
**Agborsangaya 2012 Canada**[61]	Cross-sectional	*Source of sample:* Alberta Health Quality Council of Alberta 2010 Patient Experience Survey<br><br>*Characteristics:* ≥18 years, 52.3% female, N=4980	Annual household income<br><br>Household composition (living with children vs. not, and living with adults vs. not)<br><br>*Data collection*: self-reported via telephone	Multimorbidity prevalence<br><br>*Data collection*: self-reported via telephone<br><br>*Definition:* “Presence of two or more chronic conditions”; *No. of conditions:* 16	Having an annual household income < $30,000 CAD associated with 2.39-fold increase in multimorbidity prevalence (95% CI 1.72-3.33) compared with those >=$100,000 CAD, after adjustment for age, sex, education and living with children. Association greater for age 25-44.<br><br>Not living with children associated with 2.11-fold increase in multimorbidity prevalence (95% CI 1.60-2.78) compared to those with children, after adjustment for age, sex, education and household income. Association greater for age 18-24 (although wide CIs) and age 65+. No evidence living with adults associated with multimorbidity e.g. for between 25-44, those not living with adults vs. with adults (OR 1.25, 95% CI 0.77-2.05).	H	M	M	L
**Agborsangaya 2013 Canada**[62]	Cross-sectional	*Source of sample:* Alberta Health Quality Council of Alberta 2012 Patient Experience Survey<br><br>*Characteristics*: ≥18 years, 55.8% female, N=4803	Annual household income<br><br>*Data collection*: self-reported via telephone	Multimorbidity prevalence<br><br>*Data collection*: self-reported via telephone<br><br>*Definition:* “Concurrent occurrence of two or more chronic conditions in the same individual”; *No. of conditions:* 16	Having an annual household income < $30,000 CAD associated with 2.9-fold increase in multimorbidity prevalence (95% CI 2.2-3.7) compared with those >=$100,000 CAD, after adjustment for age, sex, education and obesity status.	H	H	M	L
**Arbelle 2014 Israel**[63]	Cross-sectional	*Source of sample:* EHRs of Macabi Healthcare Service, who are legally obliged to insure every citizen. Anyone alive and member of MHS on 6th August 2012 included.<br><br>*Characteristics:* 0-85+ years, 51.2% female, N=1,972,798	Area socioeconomic deprivation<br><br>*Data collection:* Participants’ postcodes assigned to deciles of poverty index defined by parameters of 1995 national census	Multimorbidity prevalence<br><br>*Data collection*: EHRs screened for conditions in clinical coding and prescription data<br><br>*Definition:* “Two or more of these morbidities in one patient”; *No. of conditions:* 40	Residing in lowest SES area associated with higher prevalence of multimorbidity, particularly between 35 and 65, compared to those in highest SES areas. Between 45-49, multimorbidity present in 42.1% of those in lowest SES and 30.6% in highest. No substantial differences in older age groups (70+), whilst between 10-14 years multimorbidity was 3.8% in lowest SES level and 4.3% in highest.	L	M	H	H
**Bahler 2015 Switzerland**[64]	Cross-sectional	*Source of sample:* Helsana group, the leading health insurer in the country. People included if insured in 2013.<br><br>*Characteristics:* ≥65 years, 57.2% female, N=229,493	Area socioeconomic situation<br><br>*Data collection:* Polling data from GFK used as a proxy of purchasing power (available net income of population) corresponding to zip code of participants	Prevalence of multiple chronic conditions<br><br>*Data collection*: EHRs screened for conditions defined measure based on ATC classification system<br><br>*Definition:* “Two or more chronic conditions in one person”; *No. of conditions:* 22	76.7% of those residing in areas with lowest purchasing power classified as having multimorbidity and 74.8% of those residing in area with highest purchasing power.	L	L	L	H
**Barnett 2012 Scotland**[22]	Cross-sectional	*Source of sample:* Clinical data from 314 GPs. Had to be alive and permanently registered	Area socioeconomic deprivation	Multimorbidity prevalence; physical-mental multimorbidity prevalence	24.1% (23.9-24.4) of those residing in areas with highest level of deprivation had multimorbidity compared to 19.5% (19.3-19.6) of those in most affluent areas. Difference seen at all ages apart from those 85 and over. Equivalent	L	L	L	H

		with a participating practice on 31st March 2007.<br><br>*Characteristics:* 0-85+ years, 50.5% female, N=1,751,841	*Data collection:* Carstairs deciles assigned to area in which patient lived	*Data collection*: EHRs screened for conditions defined using Read Codes and prescription data<br><br>*Definition:* "Two or more morbidities in one patient"; *No. of conditions:* 40	prevalence of multimorbidity occurs 10-15 years earlier in most deprived vs. most affluent areas.<br><br>11.0% (10.9-11.2) of those residing in areas with highest level of deprivation had physical-mental multimorbidity compared to 5.9% (5.8-6.0) of those in most affluent areas.				
**Cantarero-Prieto 2018 Multi-country[70]**	Prospective cohort	*Source of sample:* 5 panel waves from Survey on Health, Ageing and Retirement in Europe. Excluded individuals who did not respond in consecutive waves<br><br>*Characteristics:* ≥50 years, 56.3% female, N=31,536	Household composition (living alone vs. not)<br><br>Rurality of household (definition unclear)<br><br>*Data collection:* interviewed (no further details)	Prevalence of multiple chronic conditions<br><br>*Data collection*: interviewed (no further details)<br><br>*Definition:* "Three or more chronic diseases"; *No. of conditions:* 14	Strong evidence that 20% higher odds of multimorbidity amongst those living alone vs. those living with others (OR=1.20, 95% CI 1.04-1.39, P<.05). Variables adjusted for unclear.<br><br>No evidence of an association between rurality of household and multimorbidity prevalence (OR 0.92, 95% CI 0.93-1.03, P>0.1). Variables adjusted for unclear.	U	U	M	U
**Cassell 2018 England[4]**	Retrospective cohort	*Source of sample:* CPRD database linked to deprivation quintiles and HES data. Included if up-to-standard registration data for at least 1 year prior to (April 16) and during study. Random subsample included.<br><br>*Characteristics:* ≥18 years, 50.7% female, N=403,985	Area socioeconomic deprivation<br><br>*Data collection:* IMD quintiles (year unclear) assigned to patient postcodes	Multimorbidity prevalence; physical-mental multimorbidity prevalence<br><br>*Data collection*: EHRs screened for conditions defined using Read Codes and product codes. 4-year lookback.<br><br>*Definition:* "Two or more currently active long-term conditions"; *No. of conditions:* 36	30.0% (29.6-30.4) of those residing in areas with highest level of deprivation had multimorbidity compared to 25.8% (25.5-26.0) of those in the most affluent areas. Difference greater for middle aged individuals (between 45 and 74 years).<br><br>14.0% (13.7-14.2) of those residing in areas with highest level of deprivation had physical-mental multimorbidity compared to 7.5% (7.2-7.7) of those in most affluent areas. Difference greater for middle aged individuals (between 35 and 84 years).	H	L	L	H
**Charlton 2013 England[34]**	Prospective cohort	*Source of sample:* CPRD database linked to deprivation quintiles. Patients with complete data on deprivation included and followed up from 1 Jan 05 - 30 April 12.<br><br>*Characteristics:* ≥ 30 years, 50% female, N=282,887	Area socioeconomic deprivation<br><br>*Data collection:* IMD quintiles (2010) assigned to patient postcode	Incidence of multiple morbidity; prevalence of depression at different levels of morbidity<br><br>*Data collection*: EHRs screened for presence of condition defined using Read Codes<br><br>*Definition:* "Dual (2 conditions) and triple (3) morbidity"; *No. of conditions:* 5	Incidence of dual and triple morbidity associated with deprivation (e.g. highest deprivation accounted for 26%, and lowest deprivation 16%, of dual condition incidences, adjusted for age and sex). Relative risk of triple morbidity was 5.51 (4.70-6.47) for most deprived quintile and 4.76 (3.81-5.96) for the least versus those developing no conditions in the least deprived quintile.<br><br>Depression was associated with deprivation at all levels of multimorbidity.	H	L	M	M
**Chung 2015 Hong Kong[65]**	Cross-sectional	*Source of sample:* Hong Kong Government's Thematic Household Survey (Oct 11-Jan 12)<br><br>*Characteristics:* ≥15 years, 52.2% female, N=25,780	Monthly household income<br><br>Household tenure<br><br>*Data collection:* self-reported using structured questionnaires given in face-to-face home interviews	Multimorbidity prevalence<br><br>*Data collection*: self-reported using structured questionnaires given in face-to-face home interviews<br><br>*Definition:* "Two or more chronic health conditions"; *No. of conditions:* 46	Reporting an income of <4,000HKD associated with 52% increased odds of multimorbidity versus reporting income of >40,000HKD (OR 1.52, 95% CI 1.39-1.66, P<.001) after adjusting for demographics, education, housing and employment status.<br><br>Compared to public (social) housing residents, homeowners, private renters and those in subsidized housing had 17% (OR 1.17, 95% CI 1.11-1.24, P=0.003), 19% (OR 1.19, 95% CI 1.09-1.29, P=0.041) and 11% (OR 1.11, 95% CI 1.05-1.18, P=0.070) higher odds of multimorbidity, respectively, in multivariate analyses.	H	M	M	L

**Foguet-Boreu 2014 Spain[36]**	Cross-sectional	*Source of sample:* EHRs collected by The Catalan Health Institute. 40% of these meet the highest quality criteria and a 2010 subsample of these used<br><br>*Characteristics:* ≥19 years, 50.7% female, N=1,749,710	Rurality of household<br><br>*Data collection:* Assigned to the participants 'area of residence' (rural if <10,000 inhabitants and/or population density <150 people/km^2, otherwise urban)	Multimorbidity prevalence<br><br>*Data collection*: EHRs screened for conditions for ICPC-2 codes classified as chronic according to O'Halloran criteria<br><br>*Definition:* "Coexistence of two or more chronic diseases"; *No. of conditions:* 146 diagnostic clusters	47.6% of those living in rural areas classified as having multimorbidity and 46.6% of those not in rural areas. Differences in prevalence similar for women and men. For example, ORs (95% CIs) for women and men (45-64 years) were 0.80 (0.78-0.82) and 0.87 (0.85-0.89), respectively (P values<.001). When stratified by age categories and adjusted for covariates, odds of multimorbidity consistently lower for those living in rural locations versus not across all age groups, although variables adjusted for unclear. Inequality in multimorbidity prevalence with area rurality greater those 45 and over for men and women.	U	L	L	U
**Hayek 2017 Israel[68]**	Cross-sectional	*Source of sample:* Israeli National Health Interview Survey (2014-2015)<br><br>*Characteristics:* ≥21 years, 49.6% female, N=4,325	Monthly household income<br><br>*Data collection:* Self-reported via questionnaire delivered via phone	Prevalence of multiple chronic conditions<br><br>*Data collection*: Self-reported over telephone if physician diagnosed them<br><br>*Definition:* "Two or more self-reported physician-diagnosed conditions"; *No. of conditions:* 10	Strong evidence that the proportion of people with multiple chronic conditions was 1.7 times higher amongst those with a monthly household income ≤$2,000 than those with >$4,000 (PRR 1.7, 95% CI 1.2-2.5, P=.005). Variables adjusted for unclear.	U	H	H	U
**Henchoz 2019 Switzerland[37]**	Retrospective cohort	*Source of sample:* Lausanne cohort 65+ study - 3 samples of population (04, 09 and 14)<br><br>*Characteristics:* 65-70 years, 58% female, N=4,055	Family economic environment (in childhood)<br><br>Household composition (living alone vs. not)<br><br>*Data collection:* Self-reported in baseline questionnaire	Multimorbidity prevalence<br><br>*Data collection*: Self-reported using questionnaire (at 2-year follow-up)<br><br>*Definition:* "Co-occurrence of two or more medical conditions"; *No. of conditions:* 13	No association between family economic environment in childhood and multimorbidity in older age (OR=0.94, 95% CI 0.74-1.19) after adjustment for sex, cohort, socioeconomic status, behaviours, other stressful events in childhood and in adulthood.<br><br>Strong evidence of an association between household composition and multimorbidity in univariate analyses (P<.001). 31.4% of those living alone classified as having multimorbidity and 24.7% of those living with others.	U	M	M	M
**Humphreys 2018 England[38]**	Prospective cohort	*Source of sample:* Hertfordshire Cohort Study, participants linked to birth records<br><br>*Characteristics:* 64-68 years, 49% female, N=1,979	Paternal social class (at birth)<br><br>*Data collection:* Nurse-administered questionnaires given during home visit at birth	Multimorbidity count<br><br>*Data collection*: Follow-up postal questionnaire asked for disease information<br><br>*Definition:* "Total number of multi-morbid conditions"; *No. of conditions:* 10	No association found between paternal social class at birth and multimorbidity count at follow-up after adjusting for baseline age, gender, health behaviours, time in cohort and year of recruitment (OR 1.15, 95% CI 0.93, 1.43, P>0.01).	H	L	H	L
**Johnson-Lawrence 2017 USA[69]**	Cross-sectional	*Source of sample:* National Health Interview Surveys (02-14). Those with education information and on or more chronic conditions included.<br><br>*Characteristics:* 30-64 years, % female N/A, N=115,097	Household income<br><br>Household tenure<br><br>*Data collection:* Self-reported in face-to-face interview	Multimorbidity prevalence<br><br>*Data collection*: Self-reported in face-to-face interview<br><br>*Definition:* "Two or more conditions"; *No. of conditions:* 9	Odds of multimorbidity increased by 45% amongst those in the bottom tertile of household income versus the highest tertile (OR 1.45, 95% 1.38-1.53) after adjusting for age, gender, ethnicity, education, interview year, region of residence, marital status, last doctor visit, employment and home ownership.<br><br>Those who rent their properties had 19% higher odds of multimorbidity compared to homeowners (OR 1.19, 95% CI 1.15-1.24) in multivariable analyses.	U	M	H	L
**Johnston 2019 Scotland[72]**	Prospective cohort	*Source of sample:* Aberdeen Children of the 50s, a cohort of individuals born in	Paternal social class (at birth)<br><br>*Data collection:* Participants' linked to birth records containing paternal occupation, coded using General Register Office's	Multimorbidity prevalence<br><br>*Data collection*: In postal questionnaire, asked to list up to six 'long-term illnesses, health problems	After adjustment for gender, educational attainment, cognition at age 7 and school type, strong evidence paternal social class at birth associated with multimorbidity in older age (P<.001). Compared to individuals who parents were in skilled manual occupations, individuals whose parents were	M	L	H	L

		Aberdeen between 1950 and 56.<br><br>*Characteristics:* age range N/A, 52.3% female, N=6,561	Occupational classification (1950)	or disabilities which limit (their) daily activities or work (they) can do'<br><br>*Definition:* "Two or more self-reported conditions"; *No. of conditions:* N/A	unemployed/disabled/dead or their occupation unknown had 74% higher odds of multimorbidity (OR 1.74, 95% CI 1.11-2.72).				
**Katikireddi 2017 Scotland**[39]	Prospective cohort	*Source of sample:* West of Scotland Twenty-07 cohort, respondents from 3 cohorts born in early 1930s, 1950s and 1970s. All cohorts and waves used in analysis apart from 1970s cohort.<br><br>*Characteristics:* 18-75 years, % female N/A, N=3,466	Household income (equivalised)<br><br>Area socioeconomic deprivation<br><br>*Data collection:* Self-reported income and weighted for no. and age of residents; Carstairs scores assigned to postcodes for deprivation	Multimorbidity prevalence<br><br>*Data collection*: Self-reported conditions in face-to-face interviews for all waves apart from wave 3 (postal questionnaire)<br><br>*Definition:* "Two or more (or three or more) of the relevant conditions"; *No. of conditions:* 40	Strong evidence found for higher odds of multimorbidity amongst those with lowest level of household income compared to highest (OR 1.53, 95% CI 1.25-1.87, P<.05). Adjusted for age, age^2, age^3, sex, cohort, prior multimorbidity, time between waves and sex*cohort interaction.<br><br>Strong evidence found for higher odds of multimorbidity amongst those living in the most deprived compared to the least deprived areas (OR 1.46, 95% CI 1.26-1.68, P<.05). Adjusted for same variables. Difference greater for those between 50 and 70 years and relationship stronger when multimorbidity defined as three or more conditions.	M	M	M	L
**Ki 2017 Korea**[40]	Longitudinal panel	*Source of sample:* Korea Health Panel Study (2009-2011, 2nd-4th waves)<br><br>*Characteristics:* ≥30 years, 53.7% female, N=9,971	Relative household poverty<br><br>*Data collection:* Self-reported income (poverty = less than half the median annual household income, equivalised to account for number of residents)	Number of diseases<br><br>*Data collection*: Self-reported in face-to-face interview/computer assisted interview. Checked using health records.<br><br>*Definition:* N/A; *No. of conditions:* 66	33% of those classified as "poor" had ≥ 3 diseases compared to 12.6% of "non-poor" participants (P<.001).	U	H	M	H
**Laires 2018 Portugal**[41]	Cross-sectional	*Source of sample:* Portuguese National Health Survey (2014)<br><br>*Characteristics:* 25-79 years, 56% female, N=15,196	Household income<br><br>*Method of data collection:* N/A	Multimorbidity prevalence<br><br>*Method of data collection*: Self-reported (no further details)<br><br>*Definition:* "Two or more of these self-reported chronic conditions"; *No. of conditions:* 13	51.2% of those with the lowest household income level were classified as having multimorbidity and 32.7% of those with the highest household income level.	L	H	M	H
**Lebenbaum 2018 Canada**[66]	Pooled cross-sectional	*Source of sample:* Pooled data from 96-97 National Population Health Survey and 05 and 12-13 Canadian Community Health Surveys<br><br>*Characteristics:* ≥18 years, 49.8% female in 96-97, 49.7% in 05, 49.6% in 12-13, N=288,300	Household income (equivalised)<br><br>Household tenure<br><br>Rurality of household (definition unclear)<br><br>*Data collection:* Self-reported via computer questionnaire. Income adjusted for no. in household.	Multimorbidity prevalence<br><br>*Data collection*: Self-reported conditions using computer assisted interview methods<br><br>*Definition:* "At least two chronic conditions"; *No. of conditions:* 10	Participants with the highest income had 43% less odds of multimorbidity compared to those with the lowest (OR 0.57, 95% CI 0.52-0.62, P<.001) after adjusting for demographic, behavioural and socioeconomic variables.<br><br>Homeownership associated with 18% lower odds of multimorbidity (OR 0.82, 95% 0.78-0.87, P<.001) in multivariate analyses.<br><br>No evidence rurality associated with multimorbidity in multivariate analyses (OR 0.98, 95% CI 0.93-1.02, P=0.323).	L	M	H	L
**Li 2016 England**[42]	Cross-sectional	*Source of sample:* Baseline data from Yorkshire Health Study.<br><br>*Characteristics:* 16-85 years, 56.3% female, N=27,806	Area socioeconomic deprivation<br><br>*Data collection:* Quintiles of IMD scores (2010) assigned to participant postcodes	Multimorbidity prevalence<br><br>*Data collection*: Self-reported conditions in questionnaire (postal or online)	45.7% of those residing in areas with the highest level of deprivation had multimorbidity compared to 26.8% of those in the most affluent areas.	M	L	M	H

				Definition: “At least two of the listed conditions”; *No. of conditions:* 12 (plus ‘other’ category)					
**Lujic 2017 Australia[43]**	Retrospective cohort	*Source of sample:* Linked data: 45 and Up Study - a random sample from Medicare data (05-09), Pharmaceutical Benefits Scheme - subsidised prescriptions (05-11), Hospital admissions data (00-13)<br><br>*Characteristics:* ≥45 years, 55.7% female, N=90,352	Household income<br><br>Speaks language other than English at home<br><br>Rurality of household (definition unclear)<br><br>*Data collection:* Self-reported in baseline questionnaire	Multimorbidity prevalence<br><br>*Data collection*: Data obtained differently depending on dataset. Self-reported in 45 and Up, EHRs screened in medication data for ICD-10-AM codes and in hospital data for ATC codes. 2-year lookback.<br><br>*Definition:* “Two or more chronic conditions”; *No. of conditions:* 8	Consistently lower odds of multimorbidity with higher income across datasets e.g. 42% lower odds if income $70k+ vs. <$20k when self-reporting health data (OR 0.58, 95% CI 0.52-0.66). Adjusted for age and sex.<br><br>Not speaking English associated with lower odds of multimorbidity in self-report data (OR 0.80, 95% CI 0.76-0.84) and higher odds in medication/hospital data (e.g. hospital: OR 1.32, 95%CI 1.32-1.42). Adjusted for age and sex.<br><br>Living in remote/very remote areas associated with increased odds of multimorbidity versus living in a major city when health information was self-reported (OR 1.14, 95% CI 1.03-1.26), obtained from medication data (OR 1.11, 95% CI 1.00-1.23) or hospital data (OR 1.28, 95% CI 1.08-1.53). Adjusted for age and sex.	H	M	M	M
**McLean 2014 Scotland[44]**	Cross-sectional	*Source of sample:* Clinical data from 314 GPs. Had to be alive and permanently registered with practice on 31st March 07.<br><br>*Characteristics:* ≥25 years, % female N/A, N=1,272,685	Area socioeconomic deprivation<br><br>*Data collection:* Carstairs deciles assigned to participant postcodes	Prevalence of physical-only multimorbidity, physical-mental multimorbidity, mental-only multimorbidity<br><br>*Data collection*: EHRs screened for presence of conditions defined using Read Codes and prescription data<br><br>*Definition:* “Coexistence of two or more chronic conditions”; *No. of conditions:* 40	14.9% of those living in most deprived areas had physical-only multimorbidity compared to 16.8% of those in the least deprived.<br><br>2.0% of those living in most deprived areas had mental-only multimorbidity compared to 0.7% of those in the least deprived. Differences greater for ages 25-54, prevalence similar ≥65 years.<br><br>17.0% of those living in most deprived areas had physical-mental multimorbidity compared to 9.0% of those in the least deprived. Differences seen in all age groups <75 years.	L	L	L	H
**Melis 2014 Sweden[45]**	Prospective cohort	*Source of sample:* Kungsholmen Project (91-93). Included those living independently and with no multimorbidity.<br><br>*Characteristics:* ≥75 years, % female N/A, N=390	Household composition (living alone vs. living with others)<br><br>*Data collection:* Self-reported in baseline social interview via standardised protocol	Multimorbidity incidence<br><br>*Data collection*: Physicians determined conditions using medical history, inpatient registry and clinical examination<br><br>*Definition:* “Co-occurrence of two or more chronic conditions”; *No. of conditions:* 38	In univariate analyses, no evidence found an association between living alone (versus with others) and multimorbidity incidence at follow-up, regardless of whether participants have no or one chronic disease at baseline (e.g. no disease at baseline OR 1.34, 95% CI 0.60-3.01).	U	M	L	H
**Moin 2018 Canada[46]**	Retrospective cross-sectional	*Source of sample:* EHRs linked to insurance data Sample contains all residents in Ontario eligible for health insurance and alive in 2015.<br><br>*Characteristics:* 0-85 years, 51.3% female, N=12,516,587	Area material deprivation<br><br>Area residential instability<br><br>Ethnic concentration of area<br><br>Area dependency (no. adults out of work or unpaid)<br><br>*Data collection:* ON-Marg Index scores assigned to postcodes	Multimorbidity prevalence<br><br>*Data collection*: EHRs screened for conditions based on ICD-10 codes. 10-year lookback.<br><br>*Definition:* “Co-occurrence of two+ (and three+) chronic conditions”; *No. of conditions:* 18	20.4% of those in the most materially deprived areas classified as having multimorbidity (2+) compared to 15.7% of those in the least. These estimates change to 9.2% and 6.3%, respectively, for multimorbidity (3+). No differences seen by gender. Differences between most and least deprived greatest for ages 55-74.<br><br>Relationship between residential instability and multimorbidity prevalence similar to material deprivation (data plotted visually but not reported in numerical form).<br><br>Ethnic concentration of residential area and dependency of residents had no association with multimorbidity prevalence (data plotted visually but not reported in numerical form).	L	L	L	H

**Mounce 2018 England**[47]	Prospective cohort	*Source of sample*: English Longitudinal Study of Ageing. Included participants in all 6 waves from 02-03 to 12-13.<br><br>*Characteristics:* ≥50 years, 56.3% female, N=5,564	Household composition (living alone vs. not)<br><br>Data collection: self-reported	Multimorbidity incidence<br><br>*Data collection*: Self-reported conditions. Ascertained at each follow-up whether mental health condition(s) in remission.<br><br>*Definition:* Two or more conditions; *No. of conditions:* 15	Living alone at baseline (versus cohabits) was not found to be associated with multimorbidity incidence after 11 years follow up (HR 0.93, 95% CI 0.71-1.21, P=.580) and after adjusting for baseline age, sex, total wealth, educational attainment, health behaviours, social detachment and locus of control.	U	M	M	L
**Neilsen 2017 Multi-country**[48]	Cross-sectional	*Source of sample:* Wave 5 of Survey on Health, Ageing and Retirement in Europe.<br><br>*Characteristics:* ≥50 years, 55.4% female, N=63,842	Monthly household income<br><br>*Data collection:* Self-reported in face-to-face interview	Multimorbidity prevalence<br><br>*Data collection*: Self-reported face-to-face<br><br>*Definition:* "Coexistence of two or more chronic conditions"; *No. of conditions:* 12	Participants with the lowest level of household income had 44% increased odds of multimorbidity compared to those with the highest (OR 1.44, 95% CI 1.32-1.59, P<.05), after adjusting for age, sex, and education level.	U	H	M	L
**Orueta 2013 Spain**[50]	Retrospective cross-sectional	*Source of sample:* EHRs from Population Stratification Programme. Included those covered by health insurance on 31st Aug 11 and for 6 months in previous year.<br><br>*Characteristics:* ≥65 years, 57.5% female, N=452,698	Area socioeconomic deprivation<br><br>*Data collection:* Participants' postcode assigned quintile of deprivation index based on census tract	Prevalence of multimorbidity (any), physical-mental, and physical only, multimorbidity<br><br>*Data collection*: EHRs screened using ACG classification system<br><br>*Definition:* "Co-occurrence of two or more (or three or more) health problems"; *No. of conditions:* 47	69.9% (69.6-70.3) of those in most deprived areas classified as having any multimorbidity vs. 60.2% (59.9-60.5) of those in the least. Inequalities greater for women and younger ages. Results similar if multimorbidity defined as 3 or more health problems.<br><br>Living in the most deprived areas (vs. the least deprived) associated with higher prevalence of physical-mental multimorbidity and physical multimorbidity (78.1% vs. 71.8%, and 62.0% vs. 51.7%, respectively).	H	L	L	H
**Orueta 2013 Spain**[49]	Retrospective cross-sectional	*Source of sample:* EHRs from Population Stratification Programme. Included those covered by health insurance on 31st Aug 11 and for 6 months in previous year.<br><br>*Characteristics:* 0-75+ years, 50.9% female, N=2,262,686	Area socioeconomic inequality<br><br>*Data collection:* Participants' postcode assigned quintile of deprivation index based on census tract. Concentration index as the measure of socioeconomic-related inequality.	Prevalence of chronic diseases<br><br>*Data collection*: EHRs screened using ACG classification system. 4-year lookback.<br><br>*Definition:* "Number of chronic conditions"; *No. of conditions:* 52	After controlling for age, individuals living in more deprived areas had disproportionately more conditions than those living in the least deprived areas. Degree of inequality increased with increasing number of conditions. Inequality was greater for females than males for all numbers of conditions.	U	L	L	H
**Orueta 2014 Spain**[51]	Retrospective cross-sectional	*Source of sample:* EHRs from Population Stratification Programme. Included those covered by health insurance on 31st Aug 11 and for 6 months in previous year.<br><br>*Characteristics:* 0-85+ years, 50.9% female, N=2,262,698	Area socioeconomic deprivation<br><br>*Method of data collection:* Participants' postcode assigned quintile of deprivation index based on census tract	Multimorbidity prevalence<br><br>*Method of data collection*: EHRs screened for presence of conditions using ACG classification system. 4-year lookback period used.<br><br>*Definition:* "Coexistence of two or more conditions in the same patient"; *No. of conditions:* 52	26.1% of those living in the most deprived areas classified as having multimorbidity compared to 20.5% of those in the least deprived. Differences greater for women than men (for women, 29.4% vs. 22.2% in most vs. least deprived areas; for men equivalent crude %s are 22.7% vs. 18.7%). Differences in prevalence as a function of area deprivation are negligible <34 years of age and most prominent between 55 and 79 years of age.	U	L	L	H
**Prazeres 2015 Portugal**[52]	Cross-sectional	*Source of sample:* Enrolled GPs who invited all adults attending consultations to participate in study during	Perceived problems managing monthly household income	Multimorbidity prevalence<br><br>*Data collection*: GPs recorded conditions using own knowledge,	No association between problems managing income and multimorbidity when defined as ≥two conditions (e.chances of multimorbidity for those self-reporting "Some monthly income left over" vs. "Not enough monthly income to make ends meet" were OR 0.8, 95% CI 0.5-1.1, P=0.182). Adjusted for age, sex,	H	M	L	L

		3 days on 3 consecutive weeks.<br><br>*Characteristics:* ≥18 years, 64.2% female, N=1,993	Household composition (living as couple, with extended family, alone or other inc. care home)<br><br>Rurality of household (definition unclear)<br><br>*Data collection:* Self-reported using questionnaire	patient's self-report and medical records<br><br>*Definition:* "Presence of ≥two or ≥three chronic health problems"; *No. of conditions:* 147 diagnostic clusters	marital status, education, professional status, residence area, living arrangement. Data not reported for ≥three.<br><br>No association found between household composition and multimorbidity in multivariate analysis. E.g. vs. living alone, ORs (95% CIs, P values) for those living as a couple were 1.4 (0.9-2.3 P=0.182 and 0.9 (0.6-1.5, P=0.778) when multimorbidity defined as 2+ and 3+ conditions, respectively, in multivariate analyses.<br><br>In multivariate analysis, residing in rural areas versus urban not associated with multimorbidity when defined as ≥two (p=0.746) or ≥three (p=0.157) conditions in multivariate analyses.				
**Roberts 2015 Canada**[53]	Cross-sectional	*Source of sample:* Canadian Community Health Survey 2011/12<br><br>*Characteristics:* ≥20 years, % female N/A, N=105,416	Household income<br><br>Highest level of education in household<br><br>Rurality of household (definition unclear)<br><br>*Data collection:* Self-reported income and education in interview	Multimorbidity prevalence<br><br>*Data collection*: Self-reported conditions on questionnaire that were "expected to last or have already lasted 6 months or more and that have been diagnosed by a health professional".<br><br>*Definition:* Two or more, and three or more, chronic diseases (3 or more used in multivariable analyses); *No. of conditions:* 9	Those in the lowest income quintile had over 4 times for odds of multimorbidity than those in the highest (OR 4.4, 95% CI 3.6-5.5), after adjusting for age, sex, household education, Aboriginal status, activity level smoking, stress, blood pressure and obesity. Difference remained across age categories, but reduced for those 65+ (OR 2.5, 95% CI 1.8-3.5)<br><br>Those living in households were no one completed high school had over 4 times odds of multimorbidity (OR 4.3, 95% CI 3.9-4.8), adjusting for same variables.<br><br>Living in rural areas associated with 10% increase in multimorbidity odds (OR 1.1, 95% CI 1.0-1.3), adjusted for age and sex.	H	M	H	M
**Ryan 2018 Canada**[54]	Cross-sectional	*Source of sample:* Linked EHRs. Participants required to be alive, have had contact with health service in 7 years and have health insurance (on 1st July 13)<br><br>*Characteristics:* 0-105 years, 50.9% female, N=13,581,191	Area material deprivation<br><br>Rurality of household (town <10,000)<br><br>*Data collection:* Quintiles of urban material deprivation-based domain of ON-Marg index assigned to participants' postcodes	Multimorbidity prevalence<br><br>*Data collection*: Presence determined if recorded in cohort and/or EHRs screened for ICD-9 or ICD-10 codes<br><br>*Definition:* "Presence of three or more chronic conditions"; *No. of conditions:* 17	Age-sex standardised rate of multimorbidity 12.3% (12.1-12.5) for those living in the most deprived urban areas and 10.3% (10.2-10.3) for those in the least deprived urban areas.<br><br>Age-sex standardised rate of multimorbidity 11.0% (11.0-11.1) for those in rural areas.	L	L	L	M
**Salisbury 2011 England**[55]	Retrospective cohort	*Source of sample:* GPRD database linked to deprivation data. Included if registered at one of practices on 1st April 05.<br><br>*Characteristics:* ≥18 years, % female N/A, N=99,997	Area socioeconomic deprivation<br><br>*Data collection:* Quintiles of Townsend calculated using census (01) data and assigned to participants' postcodes	Multimorbidity prevalence<br><br>*Data collection*: EHRs screened<br><br>*Definition:* "More than one chronic condition"; *No. of conditions:* 17 (plus ACG/EDC approach of 114 clusters)	Those in most deprived quintile for deprivation were more than twice as likely to have multimorbidity as those in the least deprived quintile (OR 1.91, 95% CI 1.78-2.04) after adjusting for age and sex.<br><br>Similar results found for ACG/EDC approach, although relationship less marked and results not shown.	U	L	L	M
**Schäfer 2012 Germany**[60]	Prospective cohort	*Source of sample:* EHRs from 158 GP practices. Included regular patients with 3 or more chronic conditions only. Exclusion criteria inc. unable to be interviewed, nursing home resident and had severe illness probably lethal in three months.	Monthly household income (equivalised)<br><br>Household tenure (owner vs. not)<br><br>Household composition (living at home alone, with spouse, with family members/others, living in assisted living/retirement home)	Multimorbidity prevalence<br><br>*Data collection*: EHRs screened for diagnoses and open questions in baseline GP interviews ("Which additional diagnoses does that patient have?").<br><br>*Definition:* Number of chronic conditions; *No. of conditions:* 29	Evidence that the number of conditions individuals have decreases by 0.27 (-0.47 to -0.08) per unit on the logarithmic scale of income (p=0.005; one step on scale equates to one of following steps: €400 to €1,100 to €3,000 to €8,100 net income per month). Adjusted for age, gender, marital status, job autonomy, household composition and tenure.<br><br>In multivariate analyse, no evidence number of chronic conditions differs with homeownership (vs. not homeowner) (-0.13 conditions, 95% CI -0.30-0.05, P=0.148) or different types of household composition (e.g. living at home with	H	M	L	U

		Characteristics: 65-84 years, 59.3% female, N=3,189	*Data collection:* Self-reported via questionnaire. Income weighted for no. and age of residents.		spouse vs. living alone associated with -0.10 conditions, 95% CI -0.42-0.23, P=0.562).				
**Sinnott 2015 Ireland**[56]	Retrospective cross-sectional	*Source of sample:* Baseline data from Mitchelstown cohort (patients from single GP).<br><br>*Characteristics:* 50-69 years, 51% female, N=2,047	Household dysfunction<br><br>*Data collection:* Self-reported during interview using ACE questionnaire	Multimorbidity prevalence, prevalence of psychiatric disease with multimorbidity<br><br>*Data collection*: Self-reported in questionnaire<br><br>*Definition:* "Two or more chronic diseases"; *No. of conditions:* 20	Higher odds of multimorbidity found for those reporting history of household dysfunction in childhood compared to those not after adjustment for age, gender, education, income, behaviour factors, depression and anxiety scores (OR 1.4, 95% CI 1.1-1.7, P<.05).<br><br>Higher odds of psychiatric disease in those with multimorbidity for those reporting household dysfunction in childhood compared to those not, after adjusting for same variables (OR 1.6, no 95% CIs).	H	M	M	L
**Stanley 2018 New Zealand**[57]	Cross-sectional	*Source of sample:* EHRs (covering all publicly funded hospital discharges, and some private, and community-dispensed prescriptions). Included individuals with health insurance (Jan 2014).<br><br>*Characteristics:* ≥18 years, 51.8% female, N=3,489,747	Area socioeconomic deprivation<br><br>*Data collection:* Quintiles of NZDep index (2013) based on NZ census and tagged to participants addresses	Multimorbidity prevalence<br><br>*Data collection*: EHRs screened for conditions. 5-year lookback for hospital data and 1 year for pharmaceutical<br><br>*Definition:* "At least two conditions from two different condition lists"; *No. of conditions:* 61 in hospital data, 30 in pharmaceutical data	Multimorbidity was more common among those in higher socioeconomic deprivation areas, with age and sex standardised prevalence based on hospital diagnoses rising from 5.8% (least deprived quintile) to 10.8% (most deprived quintile); and for pharmaceutical-based definitions from 25.1% (least deprived) to 30.9% (most deprived). Difference in prevalence with levels of deprivation greater for those aged 35-74 years old.	L	L	L	M
**Stokes 2018 New Zealand**[67]	Cross-sectional	*Source of sample:* EHRs of Maori and Pacific patients at a large urban GP in an island of NZ<br><br>*Characteristics:* ≥35 years, % female N/A, N=232	Area socioeconomic deprivation<br><br>*Data collection:* Quintiles of NZDep index tagged to participants addresses	Multimorbidity prevalence<br><br>*Data collection*: EHRs screened for conditions<br><br>*Definition:* "Presence of two of more morbidities in one patient"; *No. of conditions:* 31	61.4% of those in areas with highest level of deprivation were classified as having multimorbidity compared to 47.2% of those living in the least deprived areas. Difference in raw percentages of multimorbidity prevalence in most versus least deprived areas greater for Pacific patients than Maori patients - Pacific: 65.0% (40.8-84.6) in most deprived and 44.4% (13.7-78.8) in least, Maori: 59.5% (42.1-75.3) in most and 48.5% (28.7-68.0) in least.	H	L	L	H
**Tomasdottir 2016 Norway**[58]	Prospective cohort	*Source of sample:* Second and third waves - 95-97 and 06-08 - of the Nord-Trondelag Health Study. 11 years follow-up.<br><br>*Characteristics:* 20-59 years, 53.7% female, N=20,365	Distrusting neighbours<br><br>*Data collection:* Self-reported using questionnaire, asked to rate agreement with "Answer with regard to your environment i.e. neighbourhood/group of farms: One cannot trust each other here"	Multimorbidity prevalence<br><br>*Data collection*: Self-reporting in face-to-face interview and clinical examination<br><br>*Definition:* "Two or more coinciding chronic diseases coinciding within the same individual"; *No. of conditions:* 17	After adjustment for age, gender, smoking, physical activity, education and current depressive symptoms, no evidence of an association between distrusting neighbours at baseline and risk of developing multimorbidity within 11 years. RR for those who "strongly agree" with statement 1.13 (95% CI 0.98-1.32) compared to those who "strongly disagree".	H	H	M	L
**Tucker-Seeley 2011 USA**[71]	Retrospective cohort	*Source of sample:* 2004 wave of The Health and Retirement Study, linked to records of lifetime earnings.<br><br>*Characteristics:* 50-75+ years, 53.6% female, N=7,305	Childhood financial hardship<br><br>*Data collection:* Self-reported in interview, asked "While you were growing up, before age 16, did financial difficulties ever cause you or your family to move to a different place?"	Multimorbidity prevalence<br><br>*Data collection:* Asked if a doctor had ever told them if they have one of the diseases<br><br>*Definition: "*Count of chronic conditions"; *No. of conditions*: 6	In the unadjusted model, the expected number of chronic conditions for those reporting childhood financial hardship was 1.11 (95% CI 1.04-1.19) times that of those not reporting childhood financial hardship. After adjustment for age, gender, race and educational attainment, this estimated number of chronic conditions reduced to 1.08 (95% CI 1.02-1.14) times greater for those reporting childhood financial hardships versus those not.	U	H	H	L

**Verest 2019 Netherlands**[59]	Cross-sectional	*Source of sample:* Baseline data of HELIUS study (2011- 2015)<br><br>*Characteristics:* 18-70 years, 42.3% female, N=22,362	Problems managing household income<br><br>*Data collection:* Self-reported using questionnaire	Multimorbidity prevalence<br><br>*Data collection*: Self-reported using questionnaire, depression ≥10 on PHQ<br><br>*Definition:* "Two or more chronic diseases"; *No. of conditions:* 21	73.6% of those with "lots of problems" and 34.2% of those with "no problems" were classified as having multimorbidity. Consistent patterns of higher odds of multimorbidity in lower SES groups for men and women in all ethnic groups, after adjustment for age. E.g. odds ratio of multimorbidity for Dutch male participants reporting lots of problems was 4.48 (2.76-7.29) and for Ghanaian males was 2.79 (1.77-4.38), when compared to those with "no problems". In women, equivalent estimates were 6.82 (4.47-10.41) and 2.60 (1.79-3.77), respectively.	H	H	M	H
**Violan 2014 Spain**[35]	Cross-sectional	*Source of sample:* EHRs collected by The Catalan Health Institute. 40% of these meet the highest quality criteria and a 2010 subsample of these used<br><br>*Characteristics:* ≥19 years, 51% female, N=1,356,761	Area socioeconomic deprivation<br><br>*Data collection:* Participants' postcode assigned quintile of deprivation index based on census tract	Multimorbidity prevalence<br><br>*Data collection*: EHRs screened for conditions based on ICPC-2 codes considered chronic<br><br>*Definition:* "Coexistence of two or more chronic conditions"; *No. of conditions:* 146 diagnostic clusters	In multivariate analysis, odds of multimorbidity prevalence were greater for those in most deprived compared to the least deprived areas (OR 1.07, 95% CI 1.05-1.09). Adjusted for age, sex, number of visits home and primary care health visits during previous 12 months and quartiles of attended population.<br><br>After adjustment for number of home and primary care health visits, and quartiles of attended population, women of all ages and men aged 25 to 65 showed a significant association (i.e. increasing deprivation associated with greater multimorbidity). For under 65s, greater variation in multimorbidity for women than men across all deprivation quintiles.	U	L	L	L

[a]EHRs=electronic health records; [b]Findings reported as in paper (i.e. we have not included our own conversions of data into ORs); [c]H=high, M=Medium, L=Low, U=Unclear.
