# Supplementary material for: Household and area-level social determinants of multimorbidity: a systematic review
Source: J Epidemiol Community Health. 2020 Nov 6;75(3):232–41. doi: 10.1136/jech-2020-214691 (PMC7892392; doi:10.1136/jech-2020-214691)
Supplement: Supplementary data [file jech-2020-214691supp003.pdf]

Appendix 3: Criteria used to assess risk of selection and information bias, and confounding

**Risk of Bias**	**High**	**Medium**	**Low**	**Unclear**
**Selection bias**				
Study population not representative of the population of interest	*(1) Sample not representative of general population e.g. convenience sampling, or low response rate with large difference sample characteristics from general population*<br><br>*OR*<br><br>*(2) (For cohort) Attrition rates depend on exposed status/different levels of exposure and no statistical analysis demonstrating low-moderate effect of differential loss to follow up on effect estimates*	*(1) Random sampling from general population with baseline inclusion criteria/sampling procedure that may be moderately associated with exposures or outcome e.g. moderate response rate with small-medium difference of sample characteristics from general population.*<br><br>*OR*<br><br>*(2) (For cohort) Attrition rates moderately differ according to exposed status/different levels of exposure, or statistical analysis demonstrates only moderate effect of differential loss to follow up on effect estimates*<br><br>*OR*<br><br>*(3) (For cohort) Attrition rates depend on exposed status/different levels of exposure but statistical analysis methods to account for attrition*	*(1) Random sampling from general population with baseline inclusion criteria/sampling procedure that should not be associated with exposures or outcome. E.g. high response rate and sample seemingly representative of general population*<br><br>*OR*<br><br>*(2) Weighted sampling strategy appropriately handled in analysis*<br><br>*(3) (For cohort) Attrition rates low and similar for exposed and unexposed cohorts or different levels of exposure, or statistical analysis demonstrates only small effect of differential loss to follow up on effect estimates*	*No/inadequate information (e.g. no inclusion criteria)*

**Information bias (Exposure)**				
Potential information biases due to ascertainment of exposure(s) that is likely to be differential according to the outcome (multimorbidity)	*Self-report in circumstances likely to be strongly associated with outcome, such as retrospective recall of childhood circumstances, without appropriate sensitivity analyses or statistical adjustments to mitigate or quantify bias*	*(1) Self-report in circumstances unlikely to be associated with outcome, such as prospective data collection or objective measurement*<br><br>*AND/OR*<br><br>*(2) Self-report method validated using administrative or official data*<br><br>*AND/OR*<br><br>*(3) Administrative or official data used for all exposures but misclassification or missingness of exposure is likely to be associated with outcome*	*Validated tool used to collect information via administrative or official data with low risk of misclassification or missingness that is associated with the outcome (e.g. Index of Multiple Deprivation scores)*	*No/inadequate information (e.g. questionnaire used unavailable)*
**Information bias (Outcome)**				
Potential biases due to ascertainment of outcome (multimorbidity) that is likely to be differential according to the exposure(s).	*(1) Self-report based on a list of fewer than 12 diseases*<br><br>*OR*<br><br>*(2) Tool to measure multimorbidity not validated or validated in a very different population than study sample*	*(1) Assessment using objective tool validated in a similar population with disease list including fewer than 12 diseases*<br><br>*OR*<br><br>*(2) Administrative data with disease list including fewer than 12 diseases*<br><br>*OR*<br><br>*(3) Self-reported outcome based on a list of at least 12 diseases, with validation using administrative/official data*	*(1) Assessment using objective tool validated in a similar population, with specified list including at least 12 diseases*<br><br>*OR*<br><br>*(2) Multimorbidity ascertained from administrative data, with specified list including at least 12 diseases*	*No/inadequate information to assess risk of bias*

**Confounding**				
Potential biases due to insufficient control for confounding	*Does not address confounding by age and sex*	*Addresses confounding by age and sex (e.g. matching by age and sex; age and sex demonstrated not to be associated with exposure of interest; age and sex considered for inclusion in the final model)*	*Addresses confounding by age and sex, and at least one other potential confounder (e.g. age, sex and at least one other potential confounder eligible for inclusion in the final model)*	*No/inadequate information to assess risk of confounding*
