# Supplementary material for: Household and area-level social determinants of multimorbidity: a systematic review
Source: J Epidemiol Community Health. 2020 Nov 6;75(3):232–41. doi: 10.1136/jech-2020-214691 (PMC7892392; doi:10.1136/jech-2020-214691)
Supplement: Supplementary data [file jech-2020-214691supp004.pdf]

Appendix 4: Key study characteristics (n=41)

First Author (Year)	Country	Participants		SDoH Exposure(s)		Multimorbidity Outcome(s)		
		No.	Age range	SDoH Investigated	Method of data collection	Definition[a]	No. of conditions	Method of data collection
Cross-sectional studies								
**Agborsangaya (2012)[61]**	Canada	4980	≥18	Household: income, composition	Self-report	Presence of 2 or more chronic conditions	16	Self-report
**Agborsangaya (2013)[62]**	Canada	4803	≥18	Household: income	Self-report	Concurrent occurrence of 2 or more chronic conditions in same individual	16	Self-report
**Arbelle (2014)[63]**	Israel	1972798	0-85+	Area-level: deprivation	Poverty index	2 or more of these morbidities in one patient	40	EHRs screened
**Bahler (2015)[64]**	Switzerland	229493	≥65	Area-level: deprivation	Polling data	2 or more chronic conditions in one person	22	EHRs screened
**Barnett (2012)[22]**	Scotland	1751841	0-85+	Area-level: deprivation	Carstairs index	2 or more morbidities in one patient	40	EHRs screened
**Chung (2015)[65]**	Hong Kong	25780	≥15	Household: income, tenure	Self-report	2 or more chronic health conditions	46	Self-report
**Foguet-Boreu (2014)[36]**	Spain	1749710	≥19	Area-level: Rurality	Assigned by researcher	Coexistence of 2 or more chronic diseases	146	EHRs screened
**Hayek (2017)[68]**	Israel	4325	≥21	Household: income	Self-report	2 or more physician-diagnosed conditions	10	Self-report
**Johnson-Lawrence (2017)[69]**	USA	115097	30-64	Household: income, tenure	Self-report	2 or more conditions	9	Self-report
**Laires (2018)[41]**	Portugal	15196	25-79	Household: income	Unclear	2 or more of these chronic conditions	13	Self-report
**Lebenbaum (2018)[66]**	Canada	288300	≥18	Household: income, tenure<br>Area-level: rurality	Self-report	At least 2 chronic conditions	10	Self-report
**Li (2016)[42]**	England	27806	16-85	Area-level: deprivation	IMD (2010)	At least 2 of the listed conditions	12 (+ ‘other’)	Self-report
**McLean (2014)[44]**	Scotland	1272685	≥25	Area-level: deprivation	Carstairs index	Coexistence of 2 or more chronic conditions	40	EHRs screened

**Moin (2018)[46]**	Canada	12516587	≥50	Area-level: deprivation	ON-Marg Index	Co-occurrence of 2+ (and 3+) chronic conditions	18	EHRs screened
**Neilsen (2017)[48]**	Multiple	63842	≥50	Household: income	Self-report	Coexistence of 2 or more chronic conditions	12	Self-report
**Orueta (2013)[50]**	Spain	452698	≥65	Area-level: deprivation	Deprivation index	Co-occurrence of 2 or more (or 3 or more) health problems	47	EHRs screened
**Orueta (2013)[49]**	Spain	2262286	0-75	Area-level: socioeconomic inequality	Deprivation index	Number of chronic conditions	52	EHRs screened
**Orueta (2014)[51]**	Spain	2262698	0-85	Area-level: deprivation	Deprivation index	Coexistence of 2 or more conditions in the same patient	52	EHRs screened
**Prazeres (2015)[52]**	Portugal	1993	≥18	Household: income, composition<br>Area-level: rurality	Self-report	Presence of ≥2 or ≥3 chronic health problems	147[a]	Self-report + EHRs screened
**Roberts (2015)[53]**	Canada	105416	≥20	Household: income, education level<br>Area-level: rurality	Self-report (unclear for rurality)	2 or more, and 3 or more, chronic diseases	9	Self-report
**Ryan (2018)[54]**	Canada	13581191	0-105	Area-level: deprivation	ON-Marg Index	Presence of 3 or more chronic conditions	17	EHRs screened
**Sinnott (2015)[56]**	Ireland	2047	50-69	Household: dysfunction in childhood (e.g. divorce)	Self-report	2 or more chronic diseases	20	Self-report
**Stanley (2018)[57]**	New Zealand	3489747	≥18	Area-level: deprivation	NZDep index (2013)	At least 2 conditions from 2 different condition lists	61 and 30	EHRs screened
**Stokes (2018)[67]**	New Zealand	232	≥35	Area-level: deprivation	NZDep index (year unclear)	Presence of 2 or more morbidities in one patient	31	EHRs screened
**Verest (2019)[59]**	Netherlands	22362	18-70	Household: income	Self-report	2 or more chronic diseases	21	Self-report
**Violan (2014)[35]**	Spain	1356761	≥19	Area-level: deprivation	Deprivation index	Coexistence of 2 or more chronic conditions	146[a]	EHRs screened
Longitudinal Studies								
**Cantarero-Prieto (2018)[70]**	Multiple	31536	:≥50	Household: composition<br>Area-level: rurality	Interviewed (no further details)	3 or more chronic diseases	14	Self-report
**Cassell (2018)[4]**	England	403985	≥18	Area-level: deprivation	IMD (year unclear)	2 or more currently active long-term conditions	36	EHRs screened
**Charlton (2013)[34]**	England	282887	≥30	Area-level: deprivation	IMD (2010)	Dual (2 conditions) and triple (3) morbidity	5	EHRs screened

**Henchoz (2019)**[37]	Switzerland	4055	65-70	Household: childhood financial hardship, composition	Self-report	Co-occurrence of 2 or more medical conditions	13	Self-report
**Humphreys (2018)**[38]	England	1979	64-68	Household: paternal social class at birth (based on occupation)	Self-report	Total number of multi-morbid conditions	10	Self-report
**Johnston (2019)**[72]	Scotland	6561	N/A	Household: paternal social class at birth (based on occupation)	Self-report	2 or more self-reported conditions	N/A	Self-report
**Katikireddi (2017)**[39]	Scotland	10083	18-75	Household: income<br><br>Area-level: deprivation	Self-report and Carstairs index	2 or more of the relevant conditions	40	Self-report
**Ki (2017)**[40]	Korea	9971	≥30	Household: relative poverty (based on income)	Self-report	Number of chronic diseases	66	Self-report
**Lujic (2017)**[43]	Australia	90352	≥45	Household: income, language<br><br>Area-level: rurality	Self-report	2 or more chronic conditions	8	Self-report and EHRs screened
**Melis (2014)**[45]	Sweden	390	≥75	Household: composition	Self-report	Co-occurrence of 2 or more chronic conditions	38	Physicians determination
**Mounce (2018)**[47]	England	5564	≥50	Household: composition	Self-report	2 or more conditions	15	Self-report
**Salisbury (2011)**[55]	England	99997	≥18	Area-level: deprivation	Townsend index	More than 1 chronic condition	17 (+114[a])	EHRs screened
**Schäfer (2012)**[60]	Germany	3189	65-84	Household: income, tenure, composition	Self-report	Number of chronic conditions	29	EHRs screened
**Tomasdottir (2016)**[58]	Norway	20365	20-59	Area-level: distrust in neighbours	Self-report	2 or more coinciding chronic diseases within the same individual	17	Self-report
**Tucker-Seeley (2011)**[71]	USA	7305	50-75+	Household: childhood financial hardship	Self-report	Count of chronic conditions	6	Self-report

Note: SDoH = social determinants of health; EHRs = electronic health records; IMD = Index of Multiple Deprivation; ON-Marg = Ontario marginalisation index; NZDep = New Zealand's deprivation index
[a]Definition(s) of multimorbidity are taken as direct quotes from each paper
